# Lack of association between multiple polymorphisms in aryl hydrocarbon receptor (*AhR*) gene and cancer susceptibility

**DOI:** 10.1186/s12199-020-00907-z

**Published:** 2020-12-05

**Authors:** He Li, Li Luo, Dan Wang, Jun Duan, Rui Zhang

**Affiliations:** 1 Department of Respiratory Medicine, The Second Affiliated Hospital of Chongqing Medical University, No. 76, Linjiang Road, Chongqing, 400010 Yuzhong District China; 2 State Key Laboratory of Trauma, Burns and Combined Injury, Department of Wound Infection and Drug, Army Medical Center (Daping Hospital), Army Medical University, No. 10 Changjiang Branch Road, Chongqing, 400042 Yuzhong District China; 3 Department of Respiratory Medicine, The First Affiliated Hospital of Chongqing Medical University, No. 1, Youyi Road, Chongqing, 400016 Yuzhong District China

**Keywords:** Aryl hydrocarbon receptor, Meta-analysis, Polymorphism, Cancer risk, Susceptibility

## Abstract

**Background:**

The aryl hydrocarbon receptor (AhR) is commonly known as an environmental sensor. Polymorphisms in *AhR* gene have been implicated in susceptibility to cancer. However, the results were controversial. This study was conducted to quantitatively summarize the association between *AhR* polymorphisms and cancer risk by meta-analysis.

**Methods:**

Relevant reports were searched in four databases (Embase, PubMed, Wanfang, and China National Knowledge Infrastructure). We used pooled odds ratio (OR) and 95% confidence interval (95% CI) to evaluate the strength of the association in both standard and cumulative meta-analysis. Subgroup and sensitivity analysis was also performed, and between-study heterogeneity and publication bias were checked.

**Results:**

A total of seventeen studies referring to three *AhR* polymorphisms (rs2066853, rs7796976, and rs2074113) were identified, and 9557 cases and 10038 controls were included. There was no statistically significant association of *AhR* rs2066853 polymorphism with cancer risk in the overall population, and the negative results were repeated in subgroup analysis by the ethnicity and cancer type. Concerning *AhR* rs7796976 or rs2074113 polymorphism, no significant correlation was detected. Moreover, these non-significant findings were stable in sensitivity analysis, and the cumulative meta-analysis indicated a trend of no significant link between this three *AhR* polymorphisms and cancer risk as more data accumulated over time.

**Conclusion:**

This meta-analysis provides evidence that the rs2066853, rs7796976, or rs2074113 polymorphism in *AhR* gene is not a susceptible predictor of cancer. Further clinical and functional investigation between *AhR* polymorphisms and cancer susceptibility are needed.

**Supplementary Information:**

The online version contains supplementary material available at 10.1186/s12199-020-00907-z.

## Introduction

It is well recognized that cancer is a major public health issue, and an estimated 18.1 million new cancer cases and 9.6 million cancer deaths occurred in 2018 globally based on the GLOBOCAN data [1]. What is worse, the rising trend of cancer incidence and mortality will not slow down [1]. The exact mechanism of carcinogenesis is complex and not fully elucidated, but it has become clear that cancer links to the interaction between genetic factors and environmental exposure, such as lifestyle and chemical contaminants. Polycyclic aromatic hydrocarbons (PAHs) belong to the common environmental contaminants, and numerous PAHs are known as carcinogens [2]. The carcinogenic risk of environmental substances depends not only on the exposure dose, but also on personal susceptibility to the carcinogens [3]. Moreover, many investigations have confirmed that malignancies have some underlying genetic commonalities, and then, the hypothesis about some common susceptibility genes for the onset of cancer has been proposed [4].

In recent years, more and more studies have focused on identifying genetic causes of cancer. The human aryl hydrocarbon receptor (*AhR*) gene is located on chromosome 7p1 5[5], whose corresponding protein is a cytoplasmic ligand-activated transcription factor [6]. AhR is commonly known as an environmental sensor [7] and could be triggered by many immunological mediators to an activator of xenobiotics metabolism [6]. Namely, *AhR* gene is involved in the carcinogenic response against environmental compounds, such as 2,3,7,8-tetrachlorodibenzo-p-dioxin (TCDD) and PAHs [7, 8]. Also, as a number of physiological AhR ligands are often formed during the adaptive and innate immune response, more and more data have described the role of *AhR* gene in tumor immune surveillance and enhanced tumorigenesis [9]. Actually, AhR protein expresses in most tissues, and increased level and activity of AhR and its nuclear localization have been detected in tumor microenvironment compared with surrounding non-malignant tissues [9]. The findings indicate that the chronic activation of *AhR* may facilitate cancer development [9, 10]. Recently, accumulating evidence has showed that *AhR* could influence the major stages of tumorigenesis, such as initiation, progression, and metastasis [11]. Taken together, *AhR* could be considered as a candidate susceptibility gene for cancer based on its biological functional role.

The *AhR* gene is highly polymorphic [12, 13], and the relationship between multiple *AhR* polymorphisms and cancer risk has been investigated [14]. Nevertheless, inconsistent results were reported in different cancers and ethnicities. Meta-analysis is a valuable quantitative means to pool data from different studies and enhance statistical power, thereby overcoming the limitations of individual studies and achieving more reliable conclusions. Although two meta-analyses of AhR polymorphisms with cancer risk have been carried out [15, 16], the following reasons impelled us to update them: (1) one previous meta-analysis merely focused on the correlation between *AhR* rs2066853 polymorphism and the risk of breast cancer using three original studies [15]; (2) several case-control, cohort, or cross-sectional studies published in recent years have not been included in either of the above meta-analyses; (3) there was no subgroup analysis of ethnicity in the previous meta-analyses [15, 16], probably because of inadequate original studies; and (4) cumulative meta-analysis, which could estimate the trend and robustness of the pooled results, has not been performed in either of the previous meta-analyses. Accordingly, in the present study, meta-analysis with the most updated data was performed to explore whether *AhR* polymorphisms confer susceptibility to cancer.

## Materials and methods

### Identification of eligible studies

We conducted a systematic search of the literature in Embase, PubMed, and two Chinese databases (Wanfang and China National Knowledge Infrastructure) to identify relevant reports until July 2020. The following search terms were used: (“AhR” or “aryl hydrocarbon receptor”) and (variants or polymorphisms or variant or polymorphism or mutation) and (cancer or tumor or neoplasm or carcinoma or oma or leukemia). Moreover, the reference lists of relevant reviews were perused to acquire the previous unfound publications. Eligible studies had to fulfill all the following inclusion criteria: (1) an unrelated case-control, cross-sectional, or cohort design was used, (2) they examined the relationship between *AhR* polymorphisms and cancer risk, and (3) the genotype and allele frequencies could be available. Accordingly, publications were removed if either of the following conditions applied: (1) reports with overlapping or duplicate data or (2) literature with no useful data such as letters, abstracts, comments, or reviews. For the studies with repeated or overlapping data, the one with most subjects was selected.

### Data extraction

Based on the above selection criteria, two authors (Li and Luo) assessed all abstracts of relevant publications and reviewed the full manuscripts of included studies, independently. The following information was collected from each included study: the first author, year of publication, original country, ethnicity of the study population, age, type of cancer, total number of cases and controls, specific *AhR* polymorphisms investigated in the original studies, and genotype frequencies. The above authors extracted and compared the required information independently and then input it into the predesigned form. Any disagreement was resolved through checking the original publications and adjudicated by a third author (Wang). Ultimately, the data came to a consensus.

### Statistical analysis

Data management and statistical analysis was performed using the Comprehensive Meta-Analysis software, version 2.2 (Biostat, Englewood, New Jersey). This meta-analysis only included the polymorphisms of *AhR* gene which were investigated in at least three studies. A chi-squared test was applied to evaluate whether genotype frequencies in controls conformed to Hardy–Weinberg equilibrium (HWE). Meta-analysis was carried out in four genetic models: (1) the allelic, (2) dominant, (3) recessive, and (4) codominant model. The point estimates of odds ratio (OR) and 95% confidence interval (95% CI) were calculated to assess the strength of the link between *AhR* polymorphisms and cancer risk. Between-study heterogeneity was checked by the *Q* test, and *P* value < 0.10 was considered statistically significant. According to the absence (*P*_*Q*_ ≥ 0.10) or presence (*P*_*Q*_ < 0.10) of heterogeneity, the summary OR was calculated with the fixed- or random-effect model, respectively. Also, *I*^2^ statistics (ranging from 0 to 100%) were adopted to quantify heterogeneity, and their values of 0%, 25%, 50%, and 75% suggest no, low, moderate, and high heterogeneity, respectively [17]. The significance of pooled OR was determined with the *Z* test. Stratification was performed based on the ethnicity and cancer type, and if the similar data from at least three studies could be available in one group, other subgroup analysis was conducted.

Sensitivity analysis was adopted to validate the credibility of the findings with two methods: (i) removing a single study each time and (ii) excluding the studies that deviate from HWE in controls. Furthermore, cumulative meta-analysis was used to test the trend and robustness of the pooled results by sorting of the included studies in the descending order of publication year. Both Egger’s regression and Begg’s rank correlation methods were employed to evaluate the publication bias. Except for *Q* test, *P* < 0.05 was defined as the criterion of statistical significance.

## Results

### Main characteristics of included studies

**Fig. 1:**
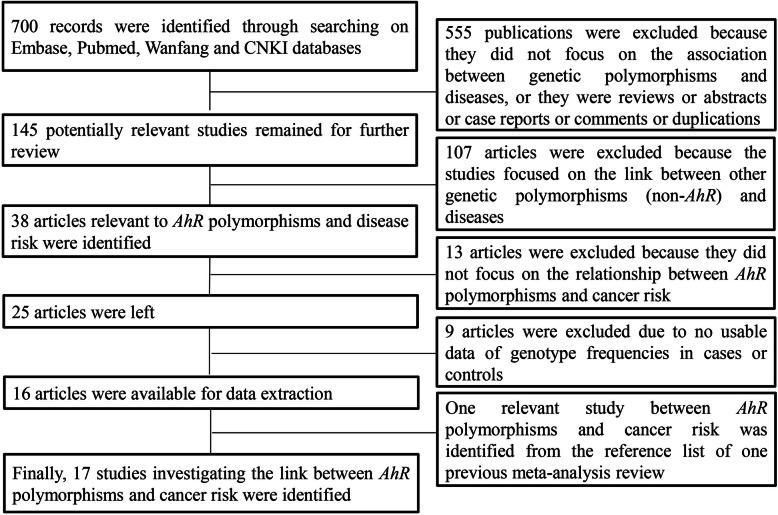
Flow chart depicting the process of identifying eligible studies

The procedure for study identification is presented in Fig. 1. Finally, a total of seventeen relevant studies were obtained through a systematic search (Table 1), including 9557 cases and 10038 controls. The included studies encompassed breast cancer (*n* = 4) [18–21], lung cancer (*n* = 6) [13, 14, 22–25], bladder cancer (*n* = 2) [5, 26], colorectal cancer (*n* = 1) [27], lymphoma (*n* = 2) [28, 29], glioma (*n* = 1) [12], and leukemia (*n* = 1) [30]. Nine studies were performed in Asians, three studies concerned Caucasians, two studies focused on South Americans, and three studies paid attention to mixed ethnicities (Table 1). Three *AhR* polymorphisms (rs2066853, rs7796976, and rs2074113) investigated in at least three original studies were identified. Disease-specific meta-analysis was carried out for lung cancer and breast cancer, and ethnicity-specific meta-analysis was performed in Chinese, Asians, and Caucasians, as there were at least three studies in the above subgroups. No other subgroup analysis was carried out due to less than three studies in the corresponding groups. The selected characteristics of the original studies and genotype distributions of *AhR* polymorphisms were illustrated in Tables 1 and 2, respectively.

**Table 1:** Characteristics of studies included in this meta-analysis

First author, year	Country	Ethnicity	Age, year (mean or range)	Cancer phenotype	Case/Control	*AhR* polymorphisms examined in the study
Zhang DS, 2002 [5]	China	Asian (Chinese)	Case:NM Con:NM	Bladder cancer	61/183	1
Gu A, 2012 [12]	China	Asian (Chinese)	Case:62.4 Con:61.5	Glioma	378/382	1, 4, 5, 8, 9, 10
Chen D, 2009 [13]	China	Asian (Chinese)	Case:59.27 Con: 59.98	Lung cancer	500/517	1, 2, 3, 4, 5, 6, 7, 8
Kawajiri K, 1995 [14]	Japan	Asian	Case:NM Con:NM	Lung cancer	321/277	1
Sangrajrang S, 2009 [18]	Thailand	Asian	Case:46.0 Con:43.2	Breast cancer	557/482	1
Sierra-Martinez M, 2018 [19]	Mexico	South American	Case:30-77 Con:30-77	Breast cancer	95/106	1
Long JR, 2006 [20]	China	Asian (Chinese)	Case:47.6 Con: 47.0	Breast cancer	1040/1099	1
Le Marchand L, 2005 [21]	USA	Mixed	Case:60.8 Con:58.3	Breast cancer	1339/1370	1
Budhwar S, 2018 [22]	India	Asian	Case:57.87 Con: 52.14	Lung cancer	297/320	1, 3, 13, 15
Cauchi S, 2001 [23]	France	Caucasian	Case:59.38 Con: 58.67	Lung cancer	177/162	1, 16
Kim JH, 2007 [24]	Korea	Asian	Case:65.3 Con:65.3	Lung cancer	616/616	1, 2, 11
Pérez-Morales R, 2014 [25]	Mexico	South American	Case:58.3 Con: 39.9	Lung cancer	190/382	1
Figueroa JD, 2008 [26]	Spanish	Caucasian	Case: 66 Con:NM	Bladder cancer	1091/1031	1, 2, 11
Cotterchio M, 2008 [27]	Canada	Caucasian	Case:20-74 Con: NM	Colorectal cancer	834/1246	1
De Roos AJ, 2006 [28]	USA	Mixed	Case:20-74 Con:20-74	Lymphoma	1128/938	1
Ng CH, 2010 [29]	Canada	Mixed	Case:20-79 Con:20-79	Lymphoma	797/791	1, 11, 12, 13, 14
Guo J, 2005 [30]	China	Asian (Chinese)	Case:≥16 Con: ≥16	Acute leukemia	136/136	1

Mixed multiple ethnicities, NM not mentioned, Con control; 1 rs2066853, 2 rs2074113, 3 rs7811989, 4 rs2158041, 5 rs1476080, 6 rs3802083, 7 rs6951212, 8 rs713150, 9 rs2106728, 10 rs6960165, 11 rs7796976, 12 rs17722841, 13 rs2282885, 14 rs17779352, 15 rs10250822, 16 ^157^(G/A)

**Table 2:** Genotype and allele distributions of *AhR* polymorphisms in cases and controls

First author, year	Sites	Cases			Controls			HWE
		GG	GA	AA	GG	GA	AA	
Zhang DS, 2002 [5]	rs2066853	29	23	9	81	75	27	Yes
Gu A, 2012 [12]		154	162	62	188	153	41	Yes
Chen D, 2009 [13]		197	205	48	222	218	49	Yes
Kawajiri K, 1995 [14]		89	174	58	94	129	54	Yes
Sangrajrang S, 2009 [18]		238	260	59	245	189	48	Yes
Sierra-Martinez M, 2018 [19]		78	16	1	82	20	4	Yes
Long JR, 2006 [20]		472	455	113	444	516	139	Yes
Le Marchand L, 2005 [21]		721	463	155	756	456	158	No
Budhwar S, 2018 [22]		229	55	13	224	67	29	No
Cauchi S, 2001 [23]		147	28	2	137	22	3	Yes
Kim JH, 2007 [24]		263	258	90	237	278	100	Yes
Pérez-Morales R, 2014 [25]		153	32	5	272	107	3	No
Figueroa JD, 2008 [26]		844	221	26	767	231	17	Yes
Cotterchio M, 2008 [27]		646	168	20	986	244	16	Yes
De Roos AJ, 2006 [28]		863	233	32	701	202	35	No
Ng CH, 2010 [29]		594	175	22	573	177	25	No
Guo J, 2005 [30]		47	61	28	74	33	29	No
		CC	CA	AA	CC	CA	AA	HWE
Chen D, 2009 [13]	rs2074113	219	228	53	240	225	52	Yes
Kim JH, 2007 [24]		265	260	85	242	207	96	No
Figueroa JD (2008) [26]		864	204	16	814	207	12	Yes
		GG	GA	AA	GG	GA	AA	HWE
Kim JH, 2007 [24]	rs7796976	280	262	74	250	280	86	Yes
Figueroa JD (2008) [26]		626	390	69	586	380	65	Yes
Ng CH, 2010 [29]		453	281	54	436	278	54	Yes

HWE Hardy–Weinberg equilibrium

### Meta-analysis of AhR rs2066853 polymorphism and cancer risk

#### Overall analysis

**Fig. 2:**
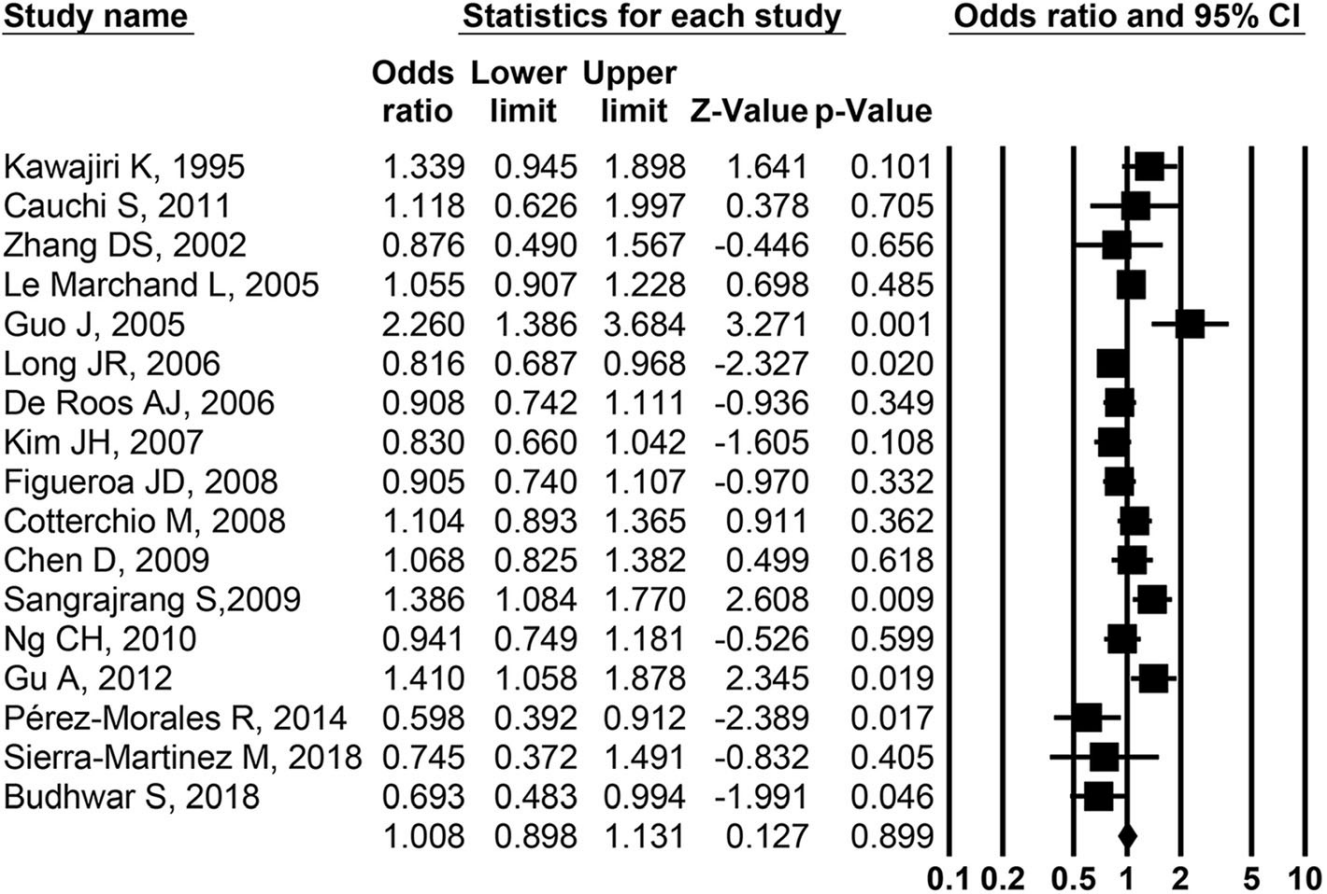
Forest plot for *AhR* rs2066853 polymorphism and the overall cancer risk in the dominant model

For *AhR* rs2066853 polymorphism, 9496 cases and 9977 controls from seventeen studies were included in meta-analysis. In the dominant model (AA+GA vs. GG), the between-study heterogeneity for all 17 studies was checked by *Q* test and the corresponding *P* value was less than 0.0001. Therefore, the random-effect model was chosen for synthesizing the data. The pooled OR for the 17 studies was 1.008 (95% CI = 0.898–1.131, *P* = 0.899; Fig. 2, Table 3), suggesting no significant link between *AhR* rs2066853 polymorphism and cancer risk in the dominant model. Similarly, combined data demonstrated no statistically significant relationship between this polymorphism and cancer risk in the recessive (Additional file 1: Fig. S1), codominant (Additional file 2: Fig. S2), or allelic model (Additional file 3: Fig. S3). All results were summarized in Table 3.

**Table 3:** Overall and subgroup meta-analysis of the association between *AhR* polymorphisms and cancer risk

Polymorphismmodel	Population	No. of studies	Test of association			Test of heterogeneity		
		(Cases/Controls)	OR	95%CI	*P*_*Z*_ value	Model	*P*_*Q*_ value	*I*^2^
rs2066853 G>A								
AA+GA vs. GG^a^	Overall	17 (9496/9977)	1.008	0.898–1.131	0.899	*R*	< 0.0001	66.662
	Asians	9 (3851/3983)	1.093	0.885–1.351	0.408	*R*	< 0.0001	78.872
	Chinese	5 (2065/2289)	1.168	0.846–1.614	0.345	*R*	< 0.0001	81.794
	Caucasians	3 (2102/2423)	1.001	0.869–1.154	0.985	*F*	0.383	0
	lung cancer	6 (2046/2245)	0.904	0.721–1.134	0.384	*R*	0.021	62.421
	breast cancer	4 (3031/3057)	1.014	0.791–1.300	0.912	*R*	0.004	77.604
AA vs. GA+GG^b^	Overall	17 (9496/9977)	0.994	0.860–1.149	0.937	*R*	0.099	32.183
	Asians	9 (3851/3983)	0.955	0.834–1.093	0.503	*F*	0.133	35.627
	Chinese	5 (2065/2289)	1.018	0.848–1.222	0.849	*F*	0.143	41.746
	Caucasians	3 (2102/2423)	1.536	0.991–2.382	0.055	*F*	0.485	0
	lung cancer	6 (2046/2245)	0.899	0.736–1.098	0.298	*F*	0.158	37.238
	breast cancer	4 (3031/3057)	0.944	0.804–1.109	0.482	*F*	0.459	0
AA vs. GG^c^	Overall	17 (9496/9977)	1.038	0.868–1.242	0.679	*R*	0.011	49.276
	Asians	9 (3851/3983)	1.024	0.797–1.315	0.856	*R*	0.007	62.256
	Chinese	5 (2065/2289)	1.157	0.791–1.694	0.452	*R*	0.014	67.815
	Caucasians	3 (2102/2423)	1.522	0.981–2.361	0.061	*F*	0.479	0
	lung cancer	6 (2046/2245)	0.903	0.728–1.120	0.351	*F*	0.102	45.628
	breast cancer	4 (3031/3057)	0.948	0.801–1.122	0.534	*F*	0.122	48.2
A vs. G^d^	Overall	17 (9496/9977)	0.995	0.911–1.086	0.906	*R*	< 0.0001	63.137
	Asians	9 (3851/3983)	1.031	0.888–1.198	0.686	*R*	< 0.0001	76.933
	Chinese	5 (2065/2289)	1.105	0.883–1.383	0.381	*R*	0.001	79.311
	Caucasians	3 (2102/2423)	1.039	0.914–1.181	0.557	*F*	0.396	0
	lung cancer	6 (2046/2245)	0.904	0.769–1.063	0.221	*R*	0.043	56.320
	breast cancer	4 (3031/3057)	0.993	0.831–1.185	0.936	*R*	0.009	73.976
rs7796976 G>A								
AA+GA vs. GG^a^	Overall	3 (2489/2415)	0.928	0.829–1.040	0.198	*F*	0.458	0.000
AA vs. GA+GG^b^	Overall	3 (2489/2415)	0.932	0.759–1.145	0.504	*F*	0.738	0.000
AA vs. GG^c^	Overall	3 (2489/2415)	0.897	0.725–1.110	0.319	*F*	0.557	0.000
A vs. G^d^	Overall	3 (2489/2415)	0.942	0.862–1.030	0.191	*F*	0.447	0.000
rs2074113 C>A								
AA+CA vs. CC^a^	Overall	3	1.021	0.895–1.165	0.755	*F*	0.613	0.000
AA vs. CA+CC^b^	Overall	3	0.895	0.706–1.135	0.361	*F*	0.275	22.596
AA vs. CC^c^	Overall	3	0.950	0.739–1.220	0.686	*F*	0.377	0.000
A vs. C^d^	Overall	3	0.992	0.893–1.102	0.876	*F*	0.574	0.000

*AhR* Aryl hydrocarbon receptor, *OR* Odds ratio, *95%CI* 95% confidence interval, *F* The fixed-effect model, *R* The random-effect model, *P*_*Z*_ *value P* value for *Z* test, *P*_*Q*_ *value P* value for *Q* test

^a^Dominant model

^b^Recessive model

^c^Codominant model

^d^Allelic model

### Subgroup analysis

**Fig. 3:**
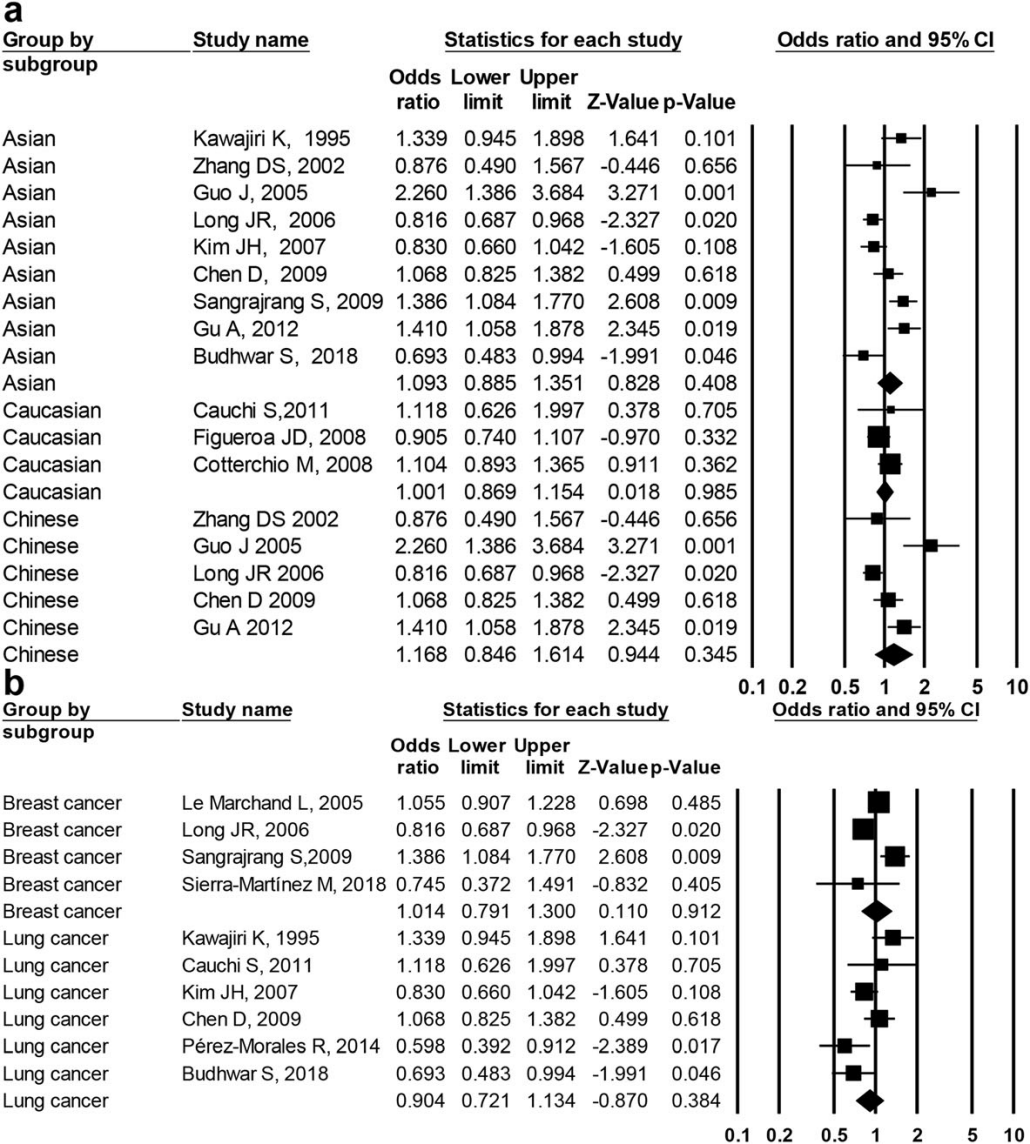
Forest plot for *AhR* rs2066853 polymorphism and the stratified cancer risk in the dominant model

Subgroup analysis by ethnicity detected the borderline association between rs2066853 polymorphism and cancer risk among Caucasians in the recessive (Additional file 4: Fig. S4a) and codominant (Additional file 5: Fig. S5a) models. Nevertheless, there was no significant association of rs2066853 polymorphism with cancer risk among Asians (AA+GA vs. GG: OR = 1.093, 95%CI = 0.885–1.351, *P* = 0.408, Fig. 3a; other models, Additional file 4, 5, and 6: Fig. S4a-S6a), Chinese (AA+GA vs. GG: OR = 1.168, 95%CI = 0.846–1.614, *P* = 0.345, Fig. 3a; other models, Additional file 4, 5, and 6: Fig. S4a-S6a)

Stratification analysis by cancer type indicated that *AhR* rs2066853 polymorphism was not significantly associated with the risk of lung cancer (AA+GA vs. GG: OR = 0.904, 95%CI = 0.721–1.134, *P* = 0.384, Fig. 3b; other models, Additional file 4, 5, and 6: Fig. S4b-S6b) or breast cancer (AA+GA vs. GG: OR = 1.014, 95%CI = 0.791–1.300, *P* = 0.912, Fig. 3b; other models, Additional file 4, 5, and 6: Fig. S4b-S6b) in any of the models. The main results of subgroup analysis were shown in Table 3.

### Meta-analysis of AhR rs7796976 and rs2074113 polymorphisms and cancer risk

**Fig. 4:**
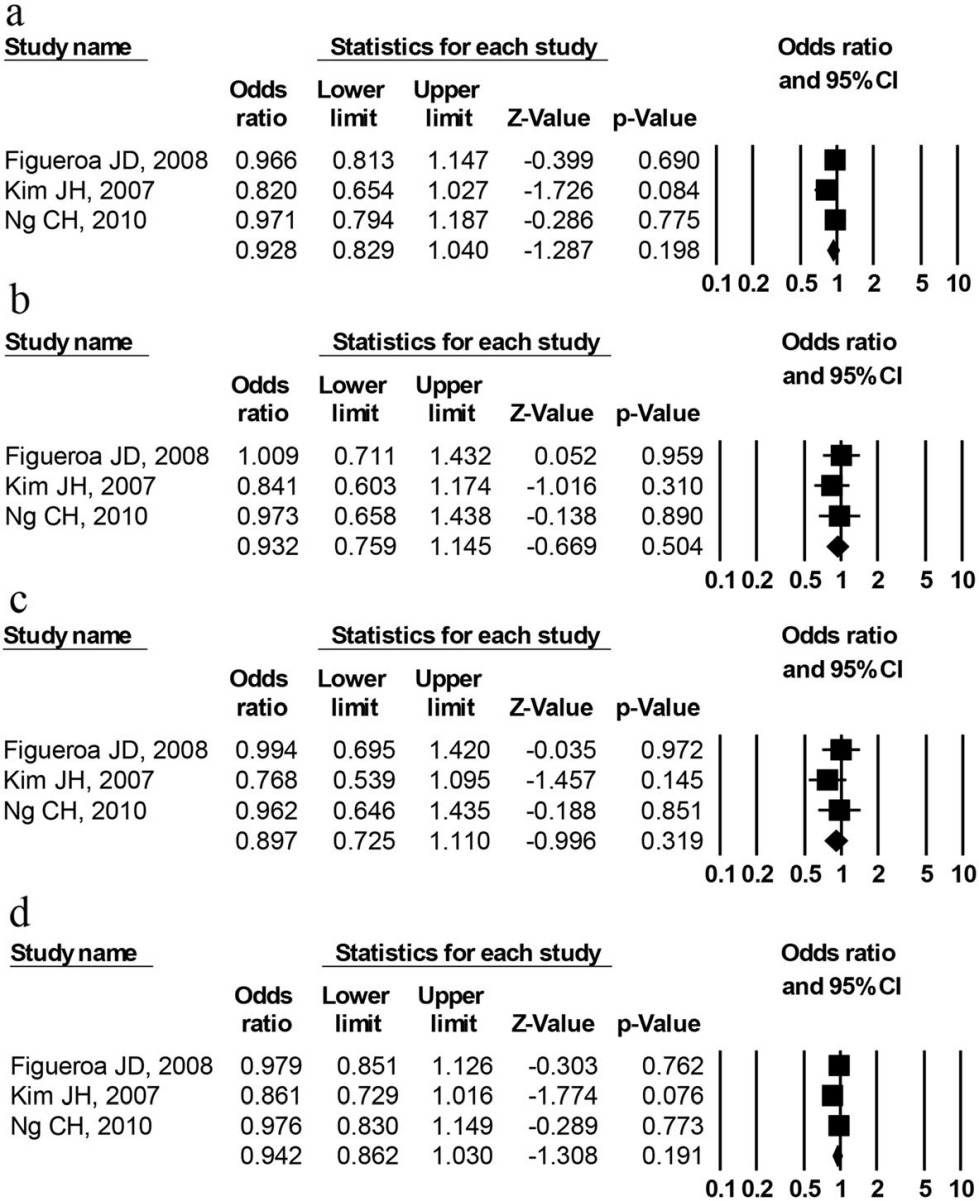
Forest plot for *AhR* rs7796976 polymorphism and the overall cancer risk. **a** Dominant model (AA+GA vs. GG), **b** recessive model (AA vs. GA+GG), **c** codominant model (AA vs. GG), and **d** allelic model (A vs. G)

**Fig. 5:**
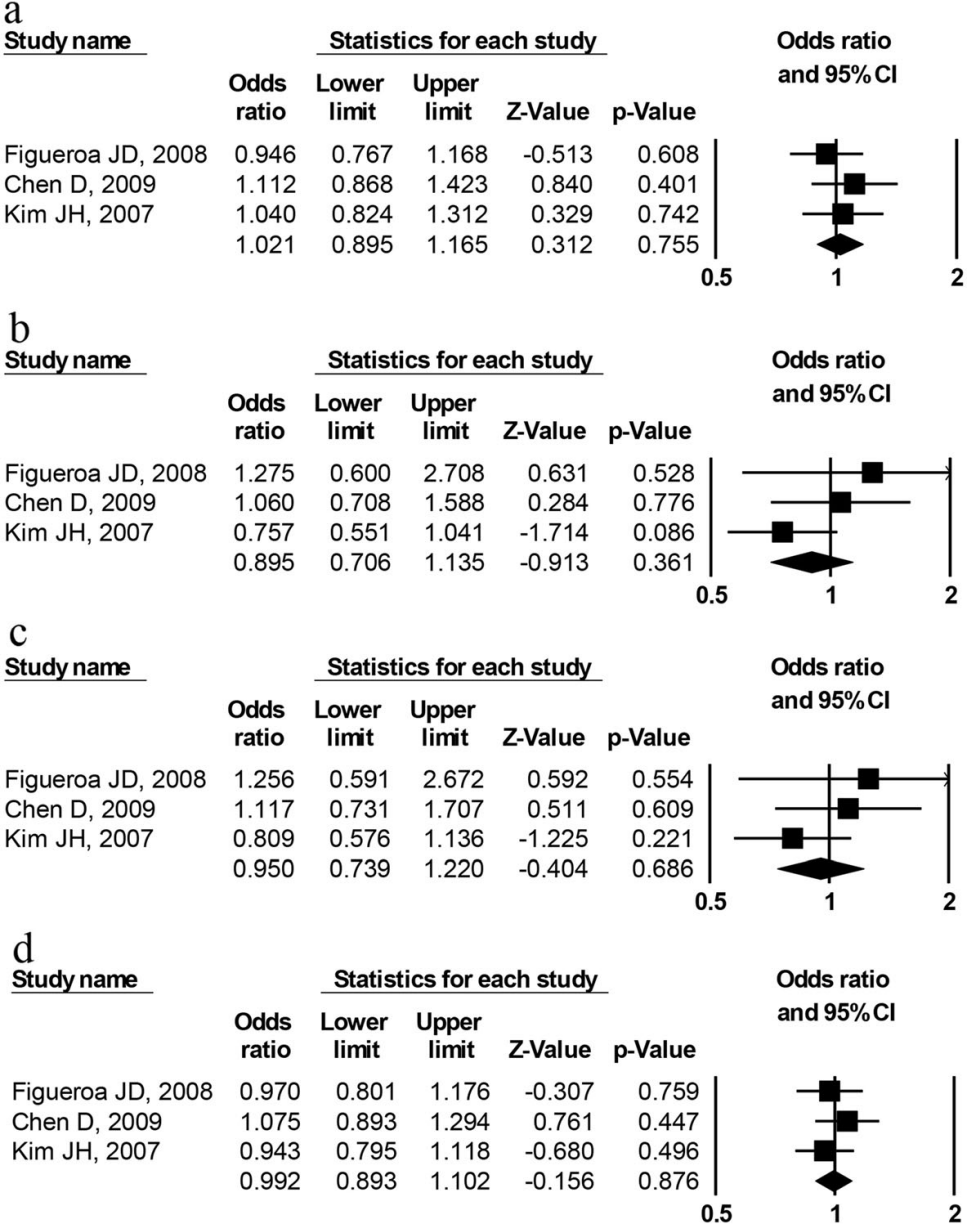
Forest plot for *AhR* rs2074113 polymorphism and the overall cancer risk. **a** Dominant model (AA+CA vs. CC), **b** recessive model (AA vs. CA+CC), **c** codominant model (AA vs. CC), and **d** allelic model (A vs. C)

There were three studies of rs7796976 polymorphism (2489 cases and 2415 controls) and three studies of rs2074113 polymorphism (2194 cases and 2095 control), respectively. The overall analysis displayed no significant association of rs7796976 (Fig. 4) or rs2074113 (Fig. 5) polymorphism with cancer risk in any of the genetic models (Table 3). For rs7796976 or rs2074113 polymorphism, no stratification analysis was carried out owing to less than three studies in any of the potential subgroups.

### Sensitivity analysis and cumulative meta-analysis

We further executed sensitivity analysis to examine the influence of individual studies on the pooled results. Regarding *AhR* rs2066853, rs7796976, or rs2074113 polymorphism, the non-significant findings in the overall or subgroup analysis were not materially altered when sequentially removing individual studies or excluding studies that deviated from HWE. Concerning the borderline association of *AhR* rs2066853 polymorphism among Caucasians in the recessive and codominant models, sensitivity analysis demonstrated that this trend was unstable and turned to no significance after excluding the study by Cotterchio et al. [27] or Figueroa et al. [26].

**Fig 6:**
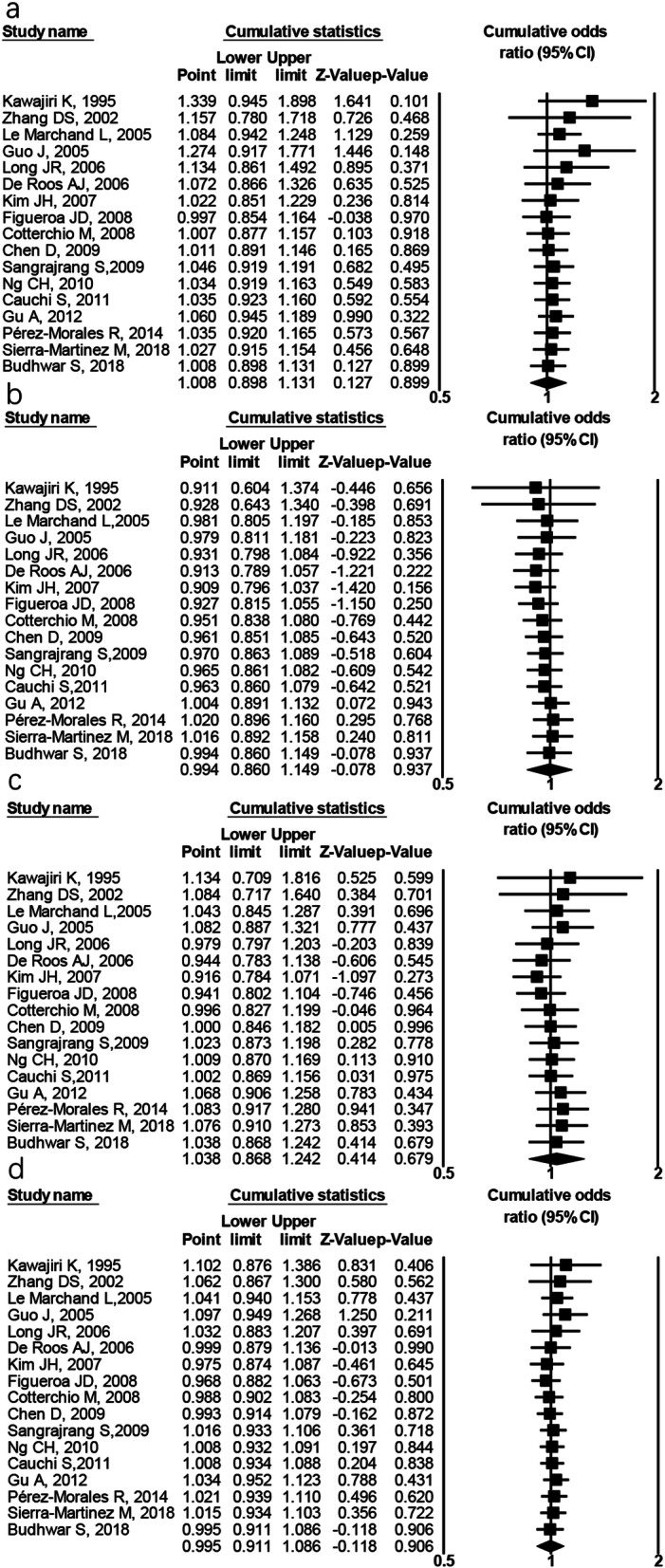
Cumulative meta-analysis for *AhR* rs2066853 polymorphism and the overall cancer risk according to publication year. **a** Dominant model (AA+GA vs. GG), **b** recessive model (AA vs. GA+GG), **c** codominant model (AA vs. GG), and **d** allelic model (A vs. G)

Cumulative analysis in the chronologic order was performed. For the relationship between *AhR* rs2066853 polymorphism and cancer risk, a stable trend of no significant results was identified among the overall sample in any of genetic models (Fig. 6). As shown in Fig. 6, the 95% CI became narrower as more data were accumulated. Furthermore, we carried out cumulative analysis in the subgroups, a stable tendency toward no significant association was repeated in the subgroups of lung cancer, breast cancer, Caucasians, Asians, or Chinese (figures not shown). With regard to the association of *AhR* rs7796976 or rs2074113 polymorphism with cancer risk, a tendency toward no significant link was also observed.

### Heterogeneity and publication bias

Between-study heterogeneity in meta-analysis was demonstrated in Table 3. For rs2066853 polymorphism, *I*^2^ statistics revealed no to high between-study heterogeneity in the overall and subgroup analysis; however, no obvious between-study heterogeneity was observed concerning rs7796976 or rs2074113 polymorphism. Neither Begg’s nor Egger’s test detected significant publication bias on *AhR* rs2066853, rs7796976, or rs2074113 polymorphism (Table 4).

**Table 4:** Publication bias results of Begg’s and Egger’s tests

	Study	Dominant model	Recessive model	Codominant model	Allelic model
Polymorphism	number (*n*)	*P*_B_ *P*_E_	*P*_B_ *P*_E_	*P*_B_ *P*_E_	*P*_B_ *P*_E_
rs2066853	17	0.650 0.663	0.967 0.799	0.711 0.664	0.773 0.942
rs2074113	3	0.296 0.019	1.000 0.386	1.000 0.428	1.000 0.689
rs7796976	3	0.296 0.401	1.000 0.593	1.000 0.721	0.296 0.567

*P*_*B*_ *P* value for Begg’s rank correlation method, *P*_*E*_ *P* value for Egger’s regression method

## Discussion

Several lines of evidence have suggested that multiple factors and multi-procedures play a crucial role in the pathogenesis of cancer, such as genetic susceptibility, environmental factors, and immunity. Intriguingly, AhR has been proven to be an environmental sensor and a key regulator of T cell differentiation on which cancer immune surveillance is dependent, so it is reasonable to infer that *AhR* gene is implicated in carcinogenesis [9, 31]. Kolluri et al. considered that AhR may be a drug target for cancer [11], and AhR inhibitors can synergize with a immunotherapeutic treatment (anti-PD-L1 antibody) to enhance antitumor activity in the mouse model of lung cancer [32]. However, *AhR* deficient mice had a more rapid progress of colon carcinoma compared to wild-type mice [8]. The above facts indicate that there is a degree of complexity and discrepancies concerning the role of *AhR* gene in malignancy, as not only protumorigenic but also antitumorigenic activities of AhR signaling have been reported [9]. Intriguingly, *AhR* is involved in inducing the transcription of several cytochrome P450 enzymes such as *CYP1A1*, which is critical in metabolizing and bioactivating environmental compounds to highly carcinogenic metabolites [9]. One previous study found that induced CYP1A1 activity was higher in the variant allele A carriers of *AhR* rs2066853(G>A) polymorphism than those with GG genotype in a Caucasian population [33]. Therefore, some polymorphisms of *AhR* gene have been suggested as potential causal variants conferring susceptibility to cancer.

On the basis of 17 eligible studies, we have conducted this comprehensive meta-analysis to evaluate the relationship between three *AhR* polymorphisms and cancer susceptibility. To the best of our knowledge, this is the first meta-analysis that investigated the association of *AhR* rs7796976 and rs2074113 sites with cancer risk. The overall analysis failed to find significant link between *AhR* rs2066853 polymorphism and cancer susceptibility (Table 3 and Fig. 2), and stratified analysis upon type of cancer and ethnicity was consistent with this negative results (Table 3 and Fig. 3). The sensitivity analysis and cumulative meta-analysis further strengthened this evidence (Fig. 6). The usage of accumulative meta-analysis and the relatively larger sample size made the current meta-analysis more precise and comprehensive, but the results of rs2066853 polymorphism were in agreement with those in the previous meta-analysis [15]. The rs2066853 site, located in the transactivation domain of *AhR* gene, could result in a nonsynonymous amino acid substitution and influence the function of AhR protein [12]. One previous study reported that the variant allele (A) of rs2066853 polymorphism had an enhanced ability in inducing the transcription of *CYP1A1* [34], but another research found that rs2066853 polymorphism did not affect the role of *AhR* gene in regulating *CYP1A1*-driven transcription [35]. Regarding rs2066853 polymorphism, conflicting evidence also existed in the studies of gene–disease association. While the mutant genotypes (AA or GA) of *AhR* rs2066853 polymorphism conferred statistically significant higher risk of glioma [12], lung cancer [13], acute leukemia [30], and breast cancer [18], some previous studies found that subjects with the same mutant genotypes (AA or GA) of rs2066853 site were more prone to have reduced risk for lung cancer [22, 25] and breast cancer [20, 25]. Thus, we speculate that no significant link between rs2066853 polymorphism and cancer risk may be ascribed to the contradictory effect of this polymorphism on the development of cancer. Actually, an estimated two thirds of cancer patients are linked to environmental factors, such as carcinogenic compounds [12], and different procarcinogens can be activated or detoxicated through phase I enzymes (mainly cytochromes P450 CYP1A1), which could be mediated by activated AhR [29]. The facts that *AhR* rs2066853 polymorphism might affect the two-way biological process (activation or detoxification) of procarcinogens regulated by CYP1A1 enzyme suggest that the mutant allele (A) and genotype (AA and GA) of this polymorphism is possibly associated with the indeterminate (increased or decreased) susceptibility to cancer, which could be used to explain the negative results in meta-analysis. Another plausible explanation of the negative findings on rs2066853 polymorphism is that the solitary polymorphism in *AhR* merely influences the transcription of *CYP1A1* gene but does not play a vital role in the subsequent pathway to cancer development.

Regarding the rs7796976 (promoter region) or rs2074113 (intronic region) polymorphism, the current meta-analysis demonstrated no significant correlation with cancer susceptibility (Figs. 4 and 5) and no obvious between-study heterogeneity indicated by *I*^2^ values (Table 3). These findings probably reflect that these polymorphisms play no role in cancer development. Noticeably, the negative results of rs7796976 or rs2074113 polymorphism should be interpreted with caution due to a small number of studies (*n* = 3). The rs7796976 (promoter region) and rs2074113 (intronic region) polymorphisms may potentially alter expression regulation of *AhR* gene and produce alternative splicing owing to the regulatory function of the intron and promoter [24, 29], but some other researchers considered that these polymorphisms could not interfere in splicing due to their distance from intron/exon junctions where splice sites are detected [29]. Actually, no effect of these two polymorphisms on *AhR* gene regulation has been revealed. It is possible that the contribution of these polymorphisms to cancer susceptibility is not great enough so that no significant correlation can be detected. More genetic association studies concerning these polymorphisms and cancer risk are warranted to validate our findings.

It is a very useful method for checking the gene-disease correlation to investigate candidate genes, but the shortcomings are inevitable. The true relationship between cancer risk and single polymorphism may be interpreted wrongly if multiple genetic variants (polymorphisms) are responsible for cancer susceptibility. The previous studies found that the combination (multiple-variant haplotype) of multiple *AhR* polymorphisms contributed to the susceptibility of lung cancer [13, 24]. Jorge-Nebert et al. constructed multiple-variant haplotypes of *AhR* gene to investigate the association of theses haplotypes with head-and-neck squamous cell carcinoma, but no *AhR* haplotypes displayed statistically significant correlation with the risk of that cancer [36]. Haplotypic analysis which has bigger statistical power than single polymorphism analysis was carried out by some original studies, but it was impossible to perform the corresponding meta-analysis of *AhR* haplotypes attributing to inadequate haplotype data. More investigations are required to evaluate the effect of *AhR* haplotypes on cancer susceptibility, which may supply the data basis for the meta-analysis of haplotypes.

Heterogeneity should be taken into account when interpreting the pooled results of *AhR* rs2066853 polymorphism in meta-analysis. Despite meticulous literature retrieval and precise data extraction, the low to moderate between-study heterogeneity was observed among the overall population (Table 3). After performing subgroup analysis by ethnicity and cancer type, the heterogeneity was not substantially reduced or eliminated apart from among the subpopulation of Caucasians. The relatively limited studies (*n* = 3) of rs2066853 polymorphism may be one reason of no obvious between-study heterogeneity in the Caucasian subgroup. Noticeably, there was low to high heterogeneity in Asian and Chinese subgroups (Table 3). Hence, we infer that the ethnicity and cancer type might not be the source of heterogeneity for rs2066853 polymorphism. The obvious between-study heterogeneity, along with the stable negative results in cumulative meta-analysis and sensitivity analysis, possibly reflects the obscure association of rs2066853 polymorphism with cancer susceptibility.

Apart from the heterogeneity, several limitations of this current meta-analysis must be addressed. First, only the published studies in limitative databases were included, so we may have missed some reports in other databases or unpublished negative investigations; additionally, the observational studies themselves were prone to publication bias. Therefore, potential publication bias could not be ruled out, even if no statistical evidence of publication bias was found in this meta-analysis (Table 4). Second, it is important to take some clinical parameters and environmental exposures into account when exploring the gene-disease association, but we failed to stratify data by age, gender, smoking status, and environmental factors due to insufficient corresponding information in the included studies. Third, the limited cases and controls in the overall or subgroup analysis may lead to relatively low statistical power and inability in detecting the low or moderate correlation. Fourth, the data of our meta-analysis were from Caucasians, Asians, and Chinese, so our findings were merely applicable to these ethnic populations. For these reasons, caution should be adopted when explaining our meta-analysis results. In order to minimize the likelihood of bias from the above limitations, we created a detailed protocol of data extraction and inclusion, conducted a comprehensive literature searching, and performed meticulous statistical analysis.

## Conclusions

In summary, this present study is the most comprehensive and latest meta-analysis concerning multiple *AhR* polymorphisms and cancer risk to date. Evidence from our meta-analysis suggests that *AhR* rs2066853 polymorphism is not a risk factor for cancer either in the overall population, Caucasians, Asians, or Chinese, and this polymorphism is not a risk factor for lung cancer or breast cancer. Our results demonstrate that *AhR* rs7796976 or rs2074113 polymorphism does not confer susceptibility to cancer. Considering the deep involvement of *AhR* gene in immune response, the influence of immune on oncogenesis, and the undeniable limitations of our meta-analysis, our findings should be viewed with caution. Future researches focusing on biological mechanisms and clinical phenotypes are required to clarify the exact role of *AhR* polymorphisms in cancer development, which may provide a sophisticated understanding of the link between *AhR* polymorphisms and cancer susceptibility.

## Supplementary Information

**Additional file 1: Fig. S1.** Forest plot for *AhR* rs2066853 polymorphism and the overall cancer risk in the recessive model.

**Additional file 2: Fig. S2.** Forest plot for *AhR* rs2066853 polymorphism and the overall cancer risk in the codominant model.

**Additional file 3: Fig. S3.** Forest plot for *AhR* rs2066853 polymorphism and the overall cancer risk in the allelic model.

**Additional file 4: Fig. S4.** Forest plot for *AhR* rs2066853 polymorphism and the stratified cancer risk in the recessive model.

**Additional file 5: Fig. S5.** Forest plot for *AhR* rs2066853 polymorphism and the stratified cancer risk in the codominant model. Asians (a), Caucasians (a), Chinese (a), breast cancer (b), and lung cancer (b).

**Additional file 6: Fig. S6.** Forest plot for *AhR* rs2066853 polymorphism and the stratified cancer risk in the allelic model.

## Data Availability

All data generated or analyzed in this study are included in this published article and its supplementary information files.

## Abbreviations

PAHs: Polycyclic aromatic hydrocarbons
AhR: Aryl hydrocarbon receptor
ARNT: AhR nuclear translocator protein
TCDD: 2,3,7,8-Tetrachlorodibenzo-p-dioxin
HWE: Hardy–Weinberg equilibrium
OR: Odds ratio
95%CI: 95% Confidence interval
