# Supplementary figures and images for: Lack of association between multiple polymorphisms in aryl hydrocarbon receptor (AhR) gene and cancer susceptibility
Source: Environ Health Prev Med. 2020 Dec 5;25:79. doi: 10.1186/s12199-020-00907-z (PMC7718691; doi:10.1186/s12199-020-00907-z)

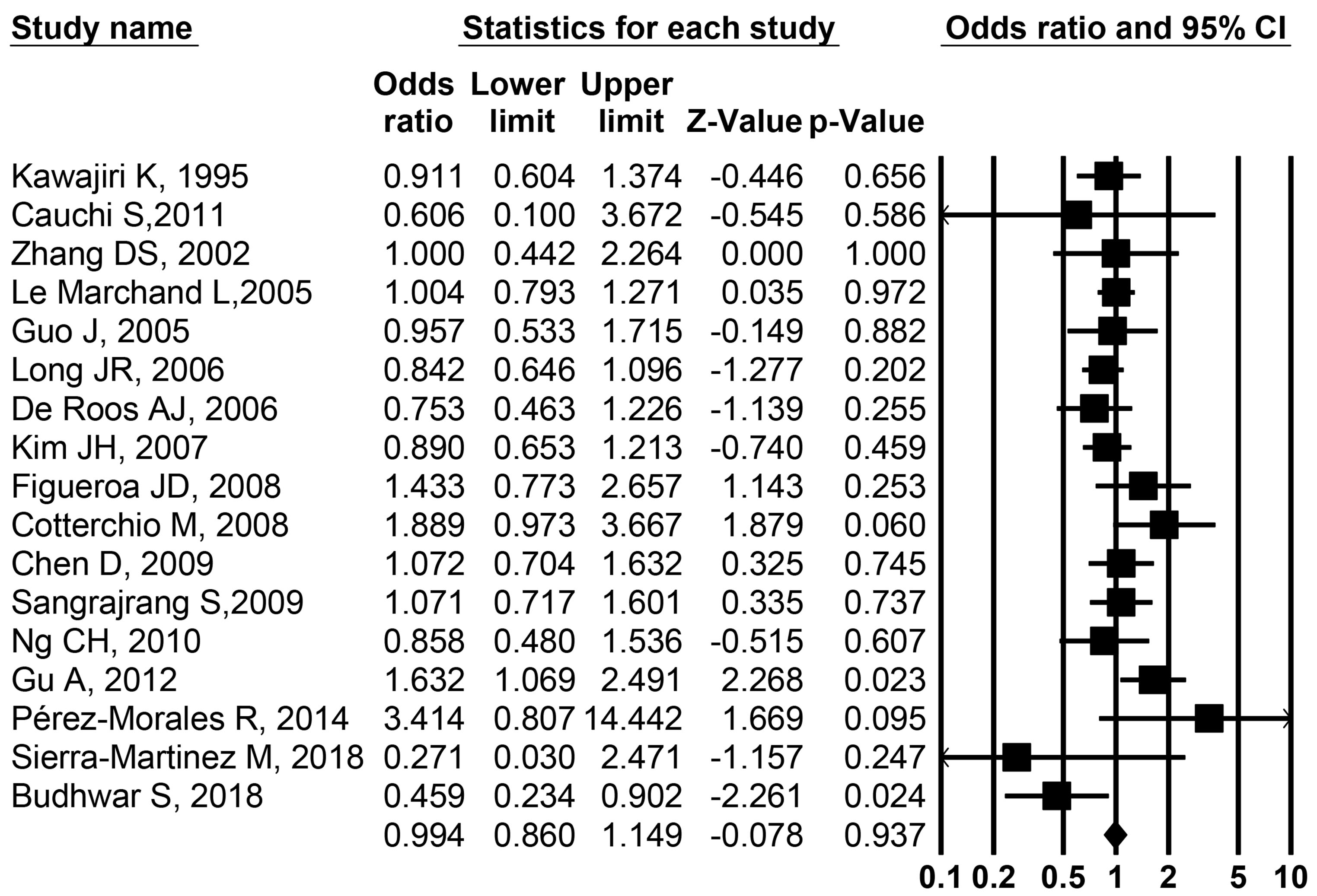

Supplement: Supplementary file 1 — Additional file 1: Fig. S1. Forest plot for AhR rs2066853 polymorphism and the overall cancer risk in the recessive model. [file 12199_2020_907_MOESM1_ESM.jpg]

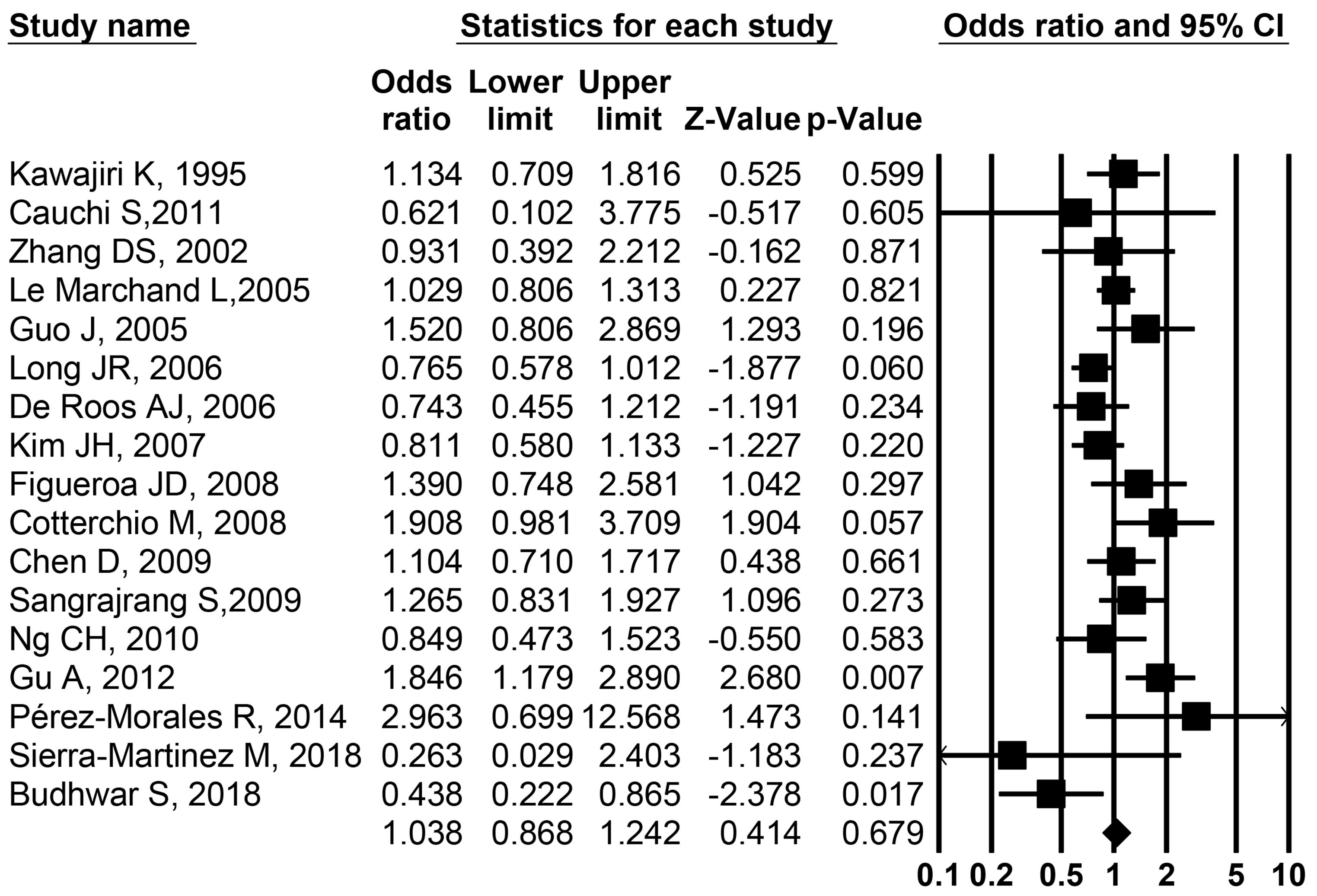

Supplement: Supplementary file 2 — Additional file 2: Fig. S2. Forest plot for AhR rs2066853 polymorphism and the overall cancer risk in the codominant model. [file 12199_2020_907_MOESM2_ESM.jpg]

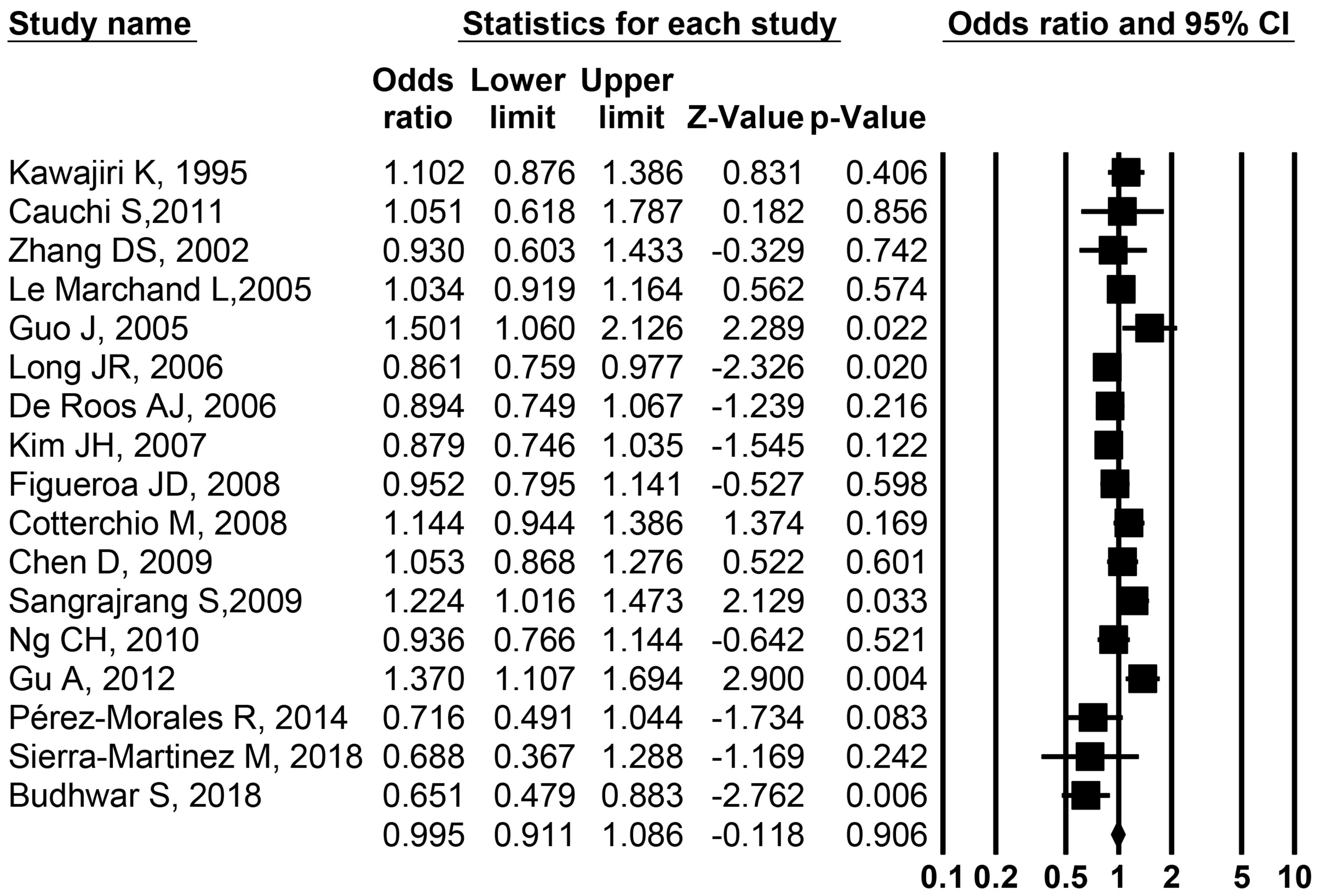

Supplement: Supplementary file 3 — Additional file 3: Fig. S3. Forest plot for AhR rs2066853 polymorphism and the overall cancer risk in the allelic model. [file 12199_2020_907_MOESM3_ESM.jpg]

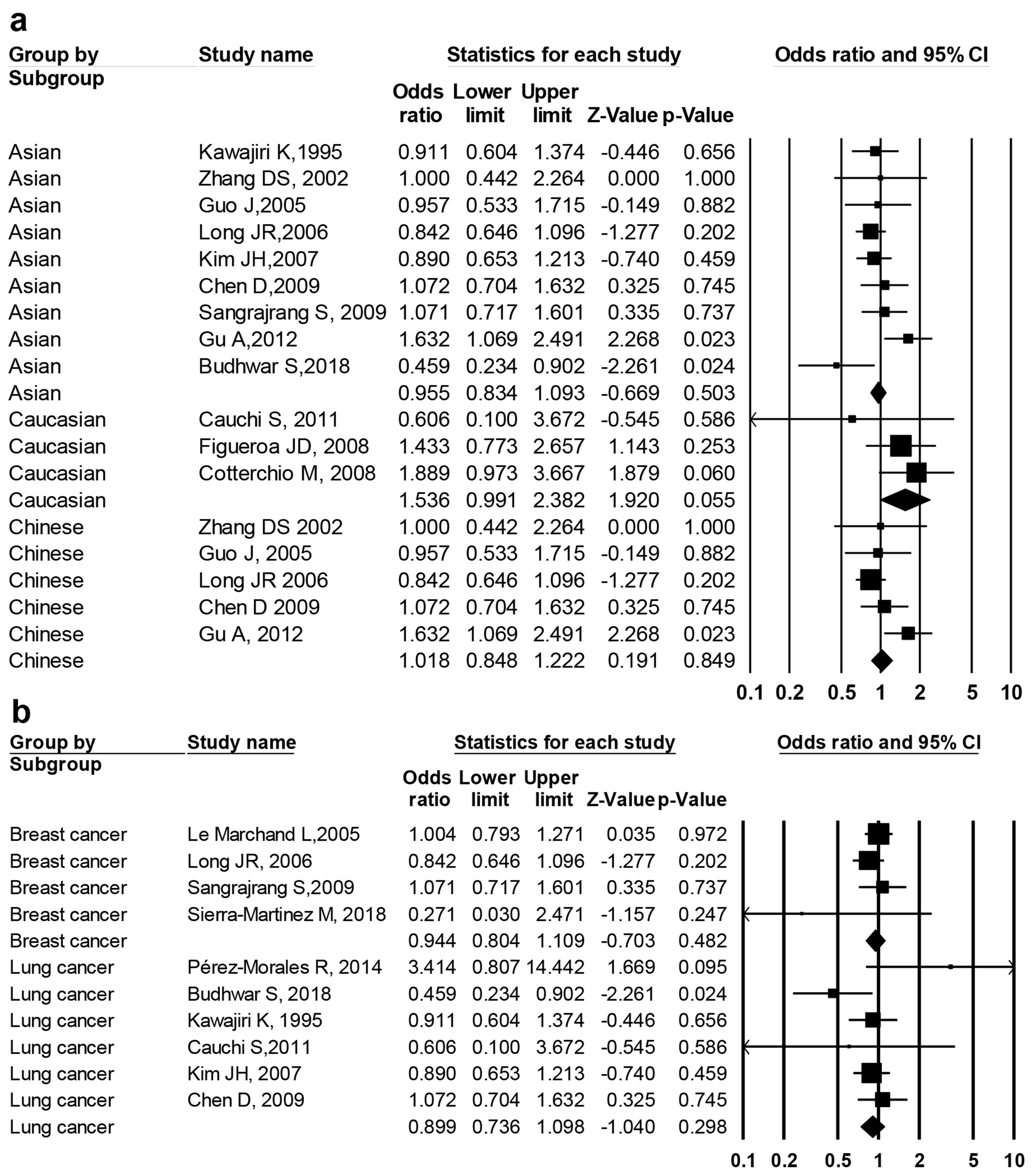

Supplement: Supplementary file 4 — Additional file 4: Fig. S4. Forest plot for AhR rs2066853 polymorphism and the stratified cancer risk in the recessive model. [file 12199_2020_907_MOESM4_ESM.jpg]

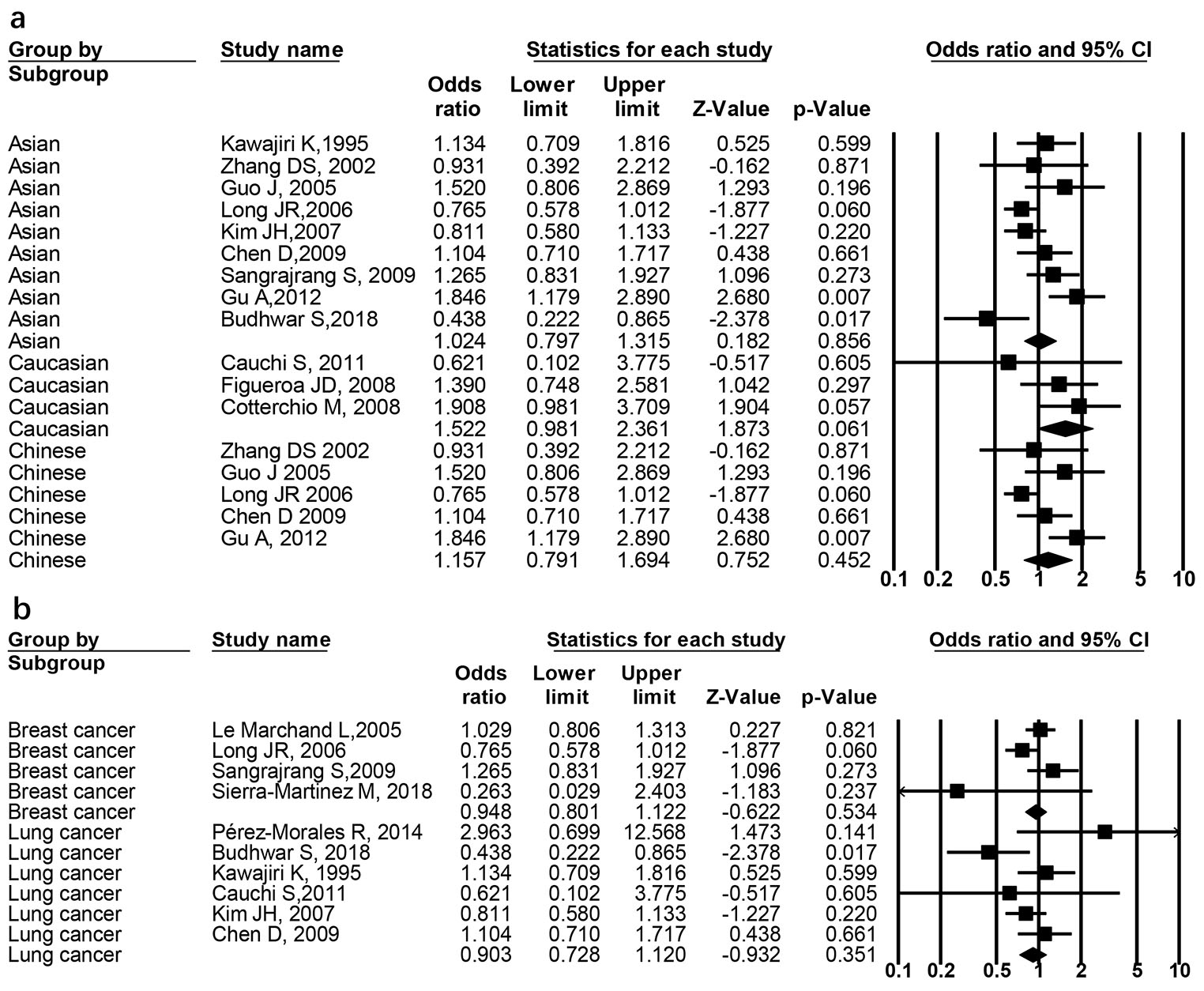

Supplement: Supplementary file 5 — Additional file 5: Fig. S5. Forest plot for AhR rs2066853 polymorphism and the stratified cancer risk in the codominant model. Asians (a), Caucasians (a), Chinese (a), breast cancer (b), and lung cancer (b). [file 12199_2020_907_MOESM5_ESM.jpg]

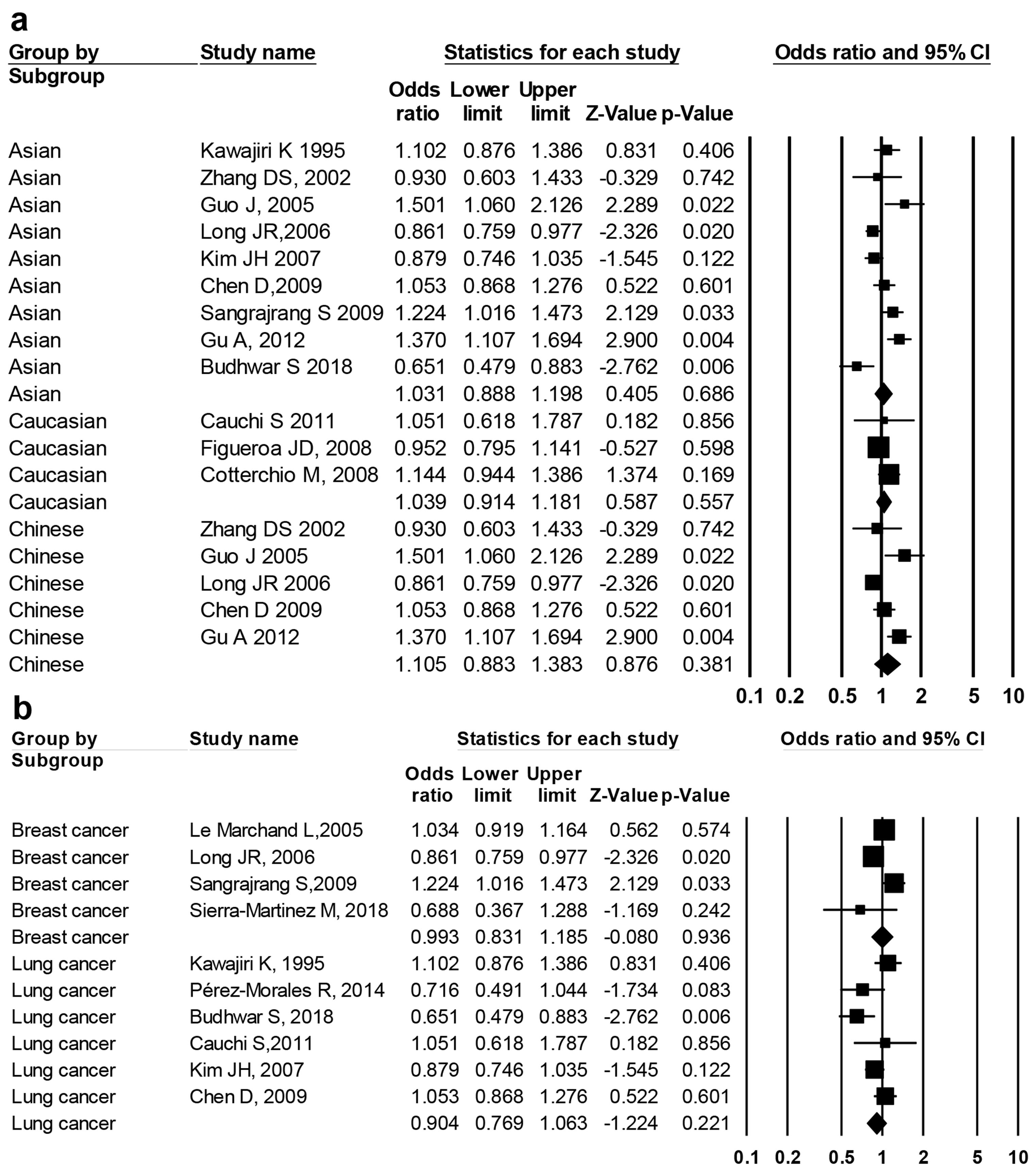

Supplement: Supplementary file 6 — Additional file 6: Fig. S6. Forest plot for AhR rs2066853 polymorphism and the stratified cancer risk in the allelic model. [file 12199_2020_907_MOESM6_ESM.jpg]
